# Exposure to dietary lipid leads to rapid production of cytosolic lipid droplets near the brush border membrane

**DOI:** 10.1186/s12986-016-0107-9

**Published:** 2016-07-28

**Authors:** Zeina Soayfane, François Tercé, Michela Cantiello, Horst Robenek, Michel Nauze, Valérie Bézirard, Sophie Allart, Bruno Payré, Florence Capilla, Christel Cartier, Christine Peres, Talal Al Saati, Vassilia Théodorou, David W. Nelson, Chi-Liang Eric Yen, Xavier Collet, Christine Coméra

**Affiliations:** 1 Institut des Maladies Métaboliques et Cardiovasculaires - I2MC, UMR 1048, Institut National de la Santé et de la Recherche Médicale, Université Toulouse III Paul Sabatier, Toulouse, F-31000 France; 2 Leibniz-Institut für Arterioskleroseforschung, Universität Münster, Münster, Germany; 3 UMR 1331 Toxalim, INRA, Université de Toulouse, ENVT, INP-Purpan, 180 chemin de Tournefeuille, BP 93173, 31027 Toulouse, cedex 3, France; 4 INSERM UMR 1043 (INSERM/UPS/CNRS/USC Inra), CHU Purpan, Toulouse, France; 5 CMEAB, Faculté de Médecine Rangueil, Toulouse, France; 6 INSERM/UPS - US006/CREFRE, Service d’Histopathologie, CHU Purpan, Toulouse, France; 7 Department of Nutritional Sciences, University of Wisconsin-Madison, Madison, WI USA

**Keywords:** Lipid droplets, Diet and dietary lipids, Intestine, Fatty acid/Metabolism, Fatty acid/Transport

## Abstract

**Background:**

Intestinal absorption of dietary lipids involves their hydrolysis in the lumen of proximal intestine as well as uptake, intracellular transport and re-assembly of hydrolyzed lipids in enterocytes, leading to the formation and secretion of the lipoproteins chylomicrons and HDL. In this study, we examined the potential involvement of cytosolic lipid droplets (CLD) whose function in the process of lipid absorption is poorly understood.

**Methods:**

Intestinal lipid absorption was studied in mouse after gavage. Three populations of CLD were purified by density ultracentrifugations, as well as the brush border membranes, which were analyzed by western-blots. Immunofluorescent localization of membranes transporters or metabolic enzymes, as well as kinetics of CLD production, were also studied in intestine or Caco-2 cells.

**Results:**

We isolated three populations of CLD (ranging from 15 to 1000 nm) which showed differential expression of the major lipid transporters scavenger receptor BI (SR-BI), cluster of differentiation 36 (CD-36), Niemann Pick C-like 1 (NPC1L1), and the ATP-binding cassette transporters ABCG5/G8 but also caveolin 2 and fatty acid binding proteins. The enzyme monoacylglycerol acyltransferase 2 (MGAT2) was identified in the brush border membrane (BBM) in addition to the endoplasmic reticulum, suggesting local synthesis of triglycerides and CLD at both places.

**Conclusions:**

We show a very fast production of CLD by enterocytes associated with a transfer of apical constituents as lipid transporters. Our findings suggest that following their uptake by enterocytes, lipids can be partially metabolized at the BBM and packaged into CLD for their transportation to the ER.

**Electronic supplementary material:**

The online version of this article (doi:10.1186/s12986-016-0107-9) contains supplementary material, which is available to authorized users.

## Background

The process of intestinal lipid absorption from lumen to blood [1–3] can be divided into four major steps. The first one concerns the emulsification and hydrolysis of dietary triglycerides, phospholipids and cholesteryl esters in the intestinal lumen through the combined actions of bile acids and digestive enzymes. The second one consists of the apical uptake of free fatty acid, monoacylglycerol, cholesterol, lysophospholipids and lipophilic vitamins by the brush border membrane (BBM) of enterocytes through transporters, such as NPC1L1 (Niemann-Pick, type C1-like 1), SR-BI (Scavenger Receptor Class BI) and CD36 (Cluster of Differentiation 36) [1, 2, 4–10]. The third step involves cytosolic transport of lipids from the BBM to the endoplasmic reticulum (ER) involving either the production of endocytic vesicules, where SR-BI and NPC1L1 have been identified [11–13], or a direct adsorption of lipids bound to the cytosolic intestinal and liver fatty acid binding protein (I-FABP and L-FABP) [14, 15]. The relative contribution of endocytosis over the non-vesicular trafficking remains unclear [16–18]. The final step is believed to start in the ER where hydrolyzed dietary lipids are metabolized into triglycerides and cholesteryl esters. While some are directed to cytosolic lipid droplets for temporary storage, these lipids are finally packaged in chylomicrons, in the ER lumen, to be secreted into the lymph and then the general circulation [1–3]. Most recently, a second pathway, involving a basolateral efflux mediated by ABCA1 and the production of intestinal HDL (iHDL) was shown to transport up to 30 % of ingested cholesterol [19].

The cytosolic lipid droplets (CLD) are produced in most eukaryotic cells and have been associated with different proteins, probably reflecting specific functions of a given cell type [20, 21]. In mammals, CLD are largely prominent in adipocytes, but also present in enterocytes and hepatocytes, especially during either feeding or fasting conditions [22–28]. Recent reports from proteomic studies have highlighted that CLD also contain proteins of the Golgi apparatus, endosomes or plasma membranes suggesting their dynamic involvement in the trafficking of both lipids and proteins between intracellular organelles [21, 23, 29, 30]. Most of lipid synthesis enzymes such as MGAT2 and diacylglycerol acyltransferases are located in the ER, the major site of lipid synthesis, but some are also transferred in the CLD of different cell types. [21–23, 25–27, 31]. This could therefore explain the presence of TG but also DG in enterocytes CLD reflecting a local fatty acid metabolism [26]. However, the exact function of CLD in enterocytes, between lipid storage, transport and metabolism still remains to be clarified [1–3, 19, 28].

In this work, we studied the early events of the uptake and accumulation of dietary lipid by enterocytes, with a particular focus on understanding the function of CLD in the flow of lipids from their absorption at the apical membrane to their packaging into lipoproteins. The droplets, previously isolated and subjected to lipidomic analysis [26], were here analyzed for their protein content. In addition, intracellular localization and movements of the CLD were examined in mouse intestine or Caco-2 cells.

## Methods

### Antibodies

We used the following antibodies: anti-SR-BI (Abcam Ab396 and Ab 3, anti-CLA1 BD bioscience), anti-CD36 (Santa Cruz H-300), anti-NPC1L1 (a homemade rabbit serum 700 against the peptide EQFHKYLPWFLNDTPNIRC and Novus NB400-128), anti-ABCG5 (Santa Cruz H-300, and rabbit antiserum generous gift of Dr. H. Hobbs), anti-ABCG8 (Santa Cruz H-300, rabbit antiserum given by H. Hobbs), anti-caveolin 2 (Santa Cruz H-96), anti-ADRP (Abcam Ab52356), anti-MGAT2 (Santa Cruz H-25), anti-DGAT1 (Abcam Ab59034), an anti-ABCA1 (Santa Cruz AB-H10), anti I-FABP (Santa Cruz C-20), anti L-FABP (Santa Cruz H-120) and anti Zonula occludens-1 (ZO-1, Invitrogen 402200).

### Mouse intestinal absorption of lipids

Animal studies were performed in conformity with the public Health Service Policy on Humane Care and Use of Laboratory Animals and in accordance to the local ethics committee (Pharmacology Toxicology, Toulouse Registered as N 86 at the Ministry of Research and Higher Education, France); notification TOXCOM 0035/EH-2013, and conducted in accordance with the European directive 2010/63/UE). C57BL/6 Rj mice from Janvier (Le-Genest-St-Isle, France) were given a standard chow diet with water *ad libitum*. Mice were fasted overnight and fed by gavage with 200 μl of 2 % (w/v) cholesterol (Sigma-Aldrich, St Quentin-Fallavier, France) in corn oil or 200 μl of H_2_O for controls. Animals were sacrificed 1 or 4 h after gavage. The proximal intestine (10 cm from the bile duct), corresponding to duodenum and proximal jejunum was removed, flushed with 5 mM sodium taurocholate (TC, Sigma) in phosphate-buffered saline (PBS) and opened. The mucosa was scraped, immediately frozen in liquid nitrogen, and stored at −80 °C.

### Intestinal lipid droplet isolation and analysis

Intestinal lipid droplets were isolated from mouse intestinal mucosa and recovered 4 h after lipid gavage, as previously described [26]. Tissues were homogenized on ice using an ultra Turrax blender in 0.8 ml of PBS supplemented with 5 mM EDTA and a cocktail of protease inhibitors (Sigma, final concentrations: 5.2 mM AEBSF (4-(2-Aminoethyl) benzenesulfonyl fluoride hydrochloride), 4 μM aprotinin, 100 μM leupeptin, 200 μM bestatin, 75 μM pepstatin A and 70 μM E-64) and was centrifuged at 250 *g* at 4 °C for 10 min to eliminate tissue debris. The remaining supernatant was named “I” for intestinal homogenate and was used to isolate three distinct CLD fractions by differential ultracentrifugations, similar to that used to purify plasma lipoproteins (TL100 apparatus, rotor TL100-4, Beckman, Villepinte, France). The first fraction D1 was isolated after centrifugation at 20 000 g for 30 min at the surface of a top cushion of PBS (d = 1 g/mL) and was referred to as chylomicron-like. Smaller droplets referred as fraction D2 were then isolated after an ultracentrifugation at 100 000 g for 1 h at d = 1. The remaining infranatant was adjusted to d = 1.21 with KBr, overlaid by a cushion of phosphate buffer at the same density and ultracentrifuged for 14 h at 120 000 g and 4 °C. The top fraction “D3” was recovered and referred to as intestinal HDL-like droplets. To efficiently purify droplets, a clear demarcation was maintained at each step, between the sample and the upper cushion, this being facilitated using concentrated homogenates. Protein concentrations were determined using Bradford reagent (Bio-Rad protein assay, Marne-La-Coquette, France). For Western-blot, samples were equilibrated in denaturing electrophoresis buffer **(**50 mM Tris/HCl, pH 6.8, 2 % SDS (w/v), 15 % glycerol (w/v), 2 % β-mercaptoethanol (v/v), 2 M Urea and 0.02 % (w/v) bromophenol blue) and were warmed for 10 min at 60 °C. Proteins were then separated on SDS-PAGE and transferred to nitrocellulose membranes. The membranes were saturated in TBS (25 mM Tris/HCl, pH = 7.4, 150 mM NaCl) plus 5 % skim milk, incubated with primary antibodies and secondary antibodies linked to the horseraddish peroxidase (HRP) and ECL reagent.

### Immuno-histochemistry

In some experiments, small pieces of proximal intestine were collected, fixed for 4 h in 4 % formaldehyde (PFA) either embedded in paraffin or frozen. Paraffin sections were regenerated by microwave in citrate buffer, pH 6, and subjected to immunodetection using horseradish peroxidase (HRP) and 3′-diaminobenzidine tetrahydrochloride (Dako). For immuno-fluorescence on frozen samples, secondary antibodies coupled to Alexafluor 488 or 594 were used.

### Flow cytometry analysis of CLD

Flow cytometry analysis was performed on intestinal isolated D1 droplets fixed in 4 % PFA for 30 min. The droplets were diluted 10 times in PBS 3 % BSA and incubated overnight with antibodies directed to either NPC1L1 or SR-BI and a secondary antibody linked to phycoerythrine (PE) or fluoresceine isothiocyanate (FITC). Samples were analyzed using a Fascalibur flow cytometer (Accuri Cytometers Inc., Ann Arbor, Michigan, USA).

### Cell culture experiments and immunofluorescence on Caco-2 cells

Caco-2 cells clone TC-7 were cultured and differentiated as previously described [9]. Mixed micelles having similar composition as natural human post-prandial micelles were prepared freshly from lipid stock solutions in chloroform/methanol (v/v, 1/1) and dried under a stream of nitrogen. The lipids were first resuspended in 208 μl DMEM 24 mM taurocholate (TC) and vigorously mixed for 2 min to form the micelles. They were diluted to 1 ml to give final concentrations of 5 mM taurocholate, 100 μM cholesterol, 500 μM oleic acid, 40 μM phosphatidylcholine, 160 μM lysophosphatidylcholine and 300 μM monooleylglycerol. The cells, previously incubated for 16 h in serum free medium, were subjected to lipid absorption from the micelles given apically up to 10 min.

After lipid absorption, apical medium was removed and the cell monolayer was fixed for 30 min in 4 % PFA and permeabilized in PBS with 0.05 % Tween 20 for 10 min or with 0.05 % saponin along with antibody incubations. Cells were saturated for 1 h in PBS 3 % BSA and incubated with primary antibodies then secondary antibodies linked to AlexaFluor 546 in PBS 1 % BSA together with fluorescent dyes to stain CLD (Bodipy 493/503 at 1 μg/ml, Molecular Probes or LD 540 0.1 μg/ml, kindly provided by C. Thiele, Dresden, Germany, 32). Cells were washed and mounted in Mowiol. Fluorescence staining was observed with a confocal microscope Zeiss LSM 510. Confocal immuno-detections were reproduced in three separate experiments with similar results.

In some experiments, the plasma membranes of living Caco-2 cells were stained using CellMask™ Deep Red (Molecular Probes) at 2.5 μg/ml for 10 min. Cells were washed 3 times in PBS, subjected to lipid absorption from apical micelles and fixed with 0.5 % (p/v) EDAC (*N*-Ethyl-*N*’-(3-dimethylaminopropyl) carbodiimide hydrochloride) as previously described [33].

Confocal imaging of CLD production, in living Caco-2 was also performed twice, with similar results, in a medium containing 1 μg/ml of LD540 in which mixed micelles were apically loaded.

In vitro absorption was also studied on isolated loops of mouse proximal intestine, incubated with luminal micelles for 1 h, followed by tissue fixation in 4 % PFA, freezing, cryosectioning and ORO staining.

### Protein biotinylation

Proteins of the apical surface of enterocytes were specifically biotinylated ex vivo*,* in a section of the proximal intestine from fasted mouse. The tissue was washed, ligated at one end, and maintained in a bath of DMEM. The intestinal lumen was filled with 0.5 mg/ml of EZ-Link Sulfo-NHS-Biotin (Pierce Biotechnology, Brebières, France) in PBS, pH 8.0 and incubated in the dark at 10 °C for 10 min, to allow surface protein biotinylation. The reaction was stopped by luminal washing with PBS plus 25 mM L-Lysine and PBS. The intestine was then filled with a DMEM solution containing mixed lipid micelles prepared as described above, and incubated for 1 h at 37 °C, then washed in PBS 5 mM TC. The mucosa was scraped, frozen and used to isolate CLD and then equilibrated in a non-denaturating buffer (without β-mercaptoethanol) for electrophoresis. Proteins were resolved as above by electrophoresis and transferred to nitrocellulose membranes. Biotinylated proteins were detected using streptavidin-HRP and ECL exposure.

### Isolation of primary enterocytes

Segments of 12 cm of proximal intestine were collected from a mouse starved for 15 h or at one hour after lipid gavage and used to isolate primary enterocytes as previously described [19]. Intestinal lumens were washed with 117 mM NaCl, 5.4 mM KCl, 0.96 mM NaH_2_PO_4_, 26.19 mM NaHCO_3_, and 5.5 mM glucose, and then, filled with 67.5 mM NaCl, 1.5 mM KCl, 0.96 mM NaH_2_PO_4_, 26.19 mM NaHCO_3_, 27 mM sodium citrate, and 5.5 mM glucose (buffer A). Intestines were then bathed in 0.9 % sodium chloride solution at 37 °C for 10 min. The buffer was discarded, and intestinal lumens were refilled with oxygenated buffer A containing 1.5 mM EDTA and 0.5 mM dithiothreitol and incubated in 0.9 % NaCl at 37 °C for 10 min. Lumenal contents were collected and enterocytes were sedimented by centrifugation at 200 g for 5 min. All buffers were adjusted to pH 7.4 and gassed with 95 % O_2_/5 % CO_2_ for 20 min prior to use.

### Freeze fracture electron microscopy

Mice were starved overnight, gavaged with 200 μl of water (control animals) or 200 μl of corn oil with 2 % cholesterol, and sacrificed 4 h later. The enterocytes of the proximal intestine were isolated as described above and prepared for freeze fracture microscopy [29]. Cells were fixed with 2 % glutaraldehyde in PBS for 2 h at room temperature, incubated in PBS 30 % glycerol for 2 h and were mounted on gold-nickel carriers and immediately frozen in Freon 22 cooled with liquid nitrogen. The samples were fractured using a freeze fracture unit (BA 310; Balzers, AG) at −100 °C. Replicas of the fractured cells were made by electron beam evaporation of platinum-carbon and carbon at angles of 38° and 90° and to a thickness of 2 nm and 20 nm, respectively. The replicas were incubated overnight in household bleach at room temperature to remove the cells. They were then washed in distilled water, mounted on grids, and examined in a scanning electron microscope (SEM, 410; Philips).

### Analysis of CLD size and purity by transmission electron microscopy: negative staining

The droplets D1, D2 and D3 were fixed in 2 % PFA, 0.1 % glutaraldehyde for 1 h and subjected to transmission electron microscopy (TEM) in order to measure their sizes and to check for eventual bilayer membranes contaminants. The CLD were mounted on formvar coated grids and incubated with uranyl acetate for 10 min, air dried for 30 min to 2 h and analyzed on MET H600 Hitashi. The D1 droplets were also sectioned by freeze fracture and analyzed by SEM.

### Epithelial membrane sub-fractionation

The apical membranes (or BBM) were separated from other membranes by the divalent cation precipitation method [34] using either scraped mucosae of the proximal intestine of starved mice for MGAT enzymatic assays or isolated enterocytes for western-blots. Briefly, tissues or cells were homogenized on ice using an ultra Turrax blender for 2 min in buffer B (300 mM mannitol, 5 mM EGTA, 10 mM Tris, pH 7.5 plus the protease inhibitors described above). The homogenate was clarified by centrifugation at 2000 *g* for 10 min, then diluted with 1 volume of H_2_0 and completed to 12 mM final of MgCl_2_. After 15 min on ice, the preparation was centrifuged at 1500 *g* for 10 min to sediment all membranes except the brush border membranes which remained in the supernatant. The microsomal pellet was washed once in buffer C (buffer B diluted to ½) containing 12 mM MgCl_2_ and centrifuged again at 1500 g for 15 min. The precipitate fraction, named Mi/Ba, contained the microsomal vesicles (ER, Golgi, intracellular vesicles) and the basolateral membranes when issued from enterocytes, but also additionally, the membranes of any other intra-mucosal cells. In both cases, the brush border membranes were separately centrifuged at 25 000 *g* for 1 h. The pellet was resuspended in buffer C plus 12 mM MgCl_2,_ incubated again for 15 min on ice and centrifuged at 1500 g for 15 min. The resulting supernatant was further centrifuged at 25 000 g for 15 min to sediment the BBM.

To analyze the purity of both membrane fractions two enzymatic assays were performed as previously described [26] measuring the activity of aminopeptidase N and alkaline phosphatase, two markers of the BBM, and the NADPH cytochrome C reductase, only expressed in the ER.

### MGAT in vitro enzymatic assay

Each reaction contained 5 mM MgCl_2_, 1.25 mg/ml BSA free fatty acid, 200 mM sucrose, 100 mM Tris HCl (pH 7.4), 50 μM acyl donor (oleoyl CoA), and 50 μM acyl acceptor 2-sn-oleoylglycerol, 2 μM orlistat (Sigma) according to Yen et al. 2003 [35]. Reactions were started by adding 100 μg of either the Mi/Ba membranes or the BBM to the assay mix, in 1 ml final and were stopped after 90 min by adding 2.5 ml of dichloromethane:methanol (1:1, v:v). The extracted lipids were dried and the molecular species of neutral lipids (cholesterol, cholesteryl esters, diacylglycerols, and triacylglycerols) were quantitated by gas liquid chromatography [36]. The CE, DG and TG produced during the assay were estimated after subtracting the neutral lipid found in control membranes (Mi/Ba or BBM) incubated in the same conditions but without oleoyl CoA and 2-sn oleolylglycerol.

## Results

### Formation of lipid droplets in the enterocytes after lipid load

**Fig. 1:**
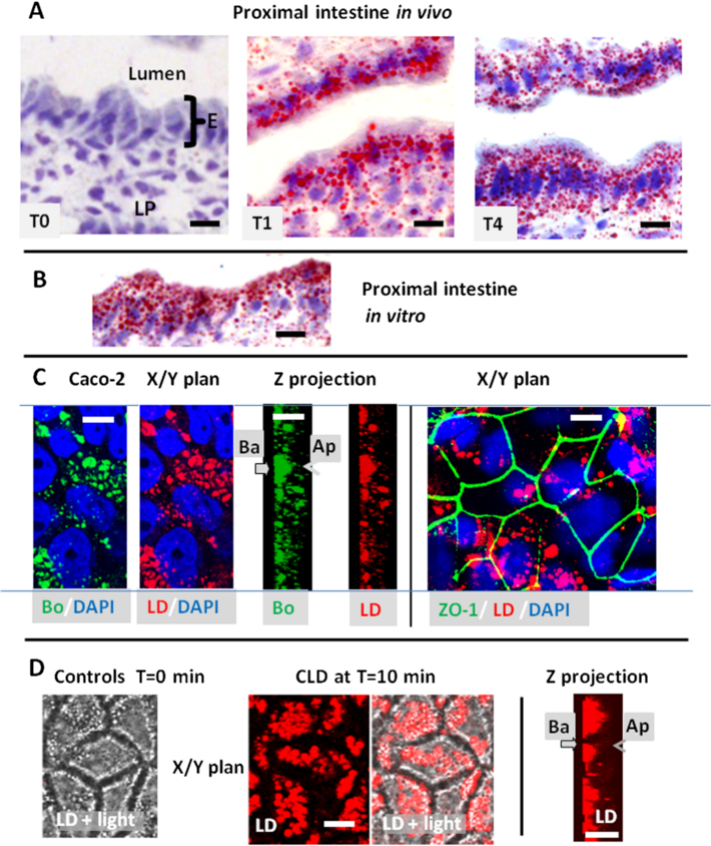
Identification of CLD in mouse intestine (**a**, **b**) or Caco-2-cells (**c**, **d**). **a, b**) Oil red O staining of CLD in the proximal intestine of mice produced (**a**) in vivo after an acute lipid gavage showing their absence in the fasted state (T0) and their abundance at 1 h (T1) or 4 h (T4) and (**b**)) in vitro after 1 h incubation of isolated intestinal loop with luminal mixed micelles. E: enterocytes, LP: lamina propria, **c**) Visualisation of CLD accumulation in differentiated Caco-2 cells fixed at 10 min after apical addition of mixed micelles, using Bodipy 494/503 (Bo) or LD540 (LD), in either a transverse plane (x/y) or the corresponding Z projection. The nuclei were stained with DAPI and the tight junction constituent ZO-1 was immunolabeled. Ap: apical and Ba: basolateral poles. Bars: 10 μM. **d**) Live imaging of Caco-2 cells, performed in the presence of LD540 in the medium and apical loading of mixed micelles, showing no CLD at T = 0 min but their accumulation in the basement of living Caco-2 cells at T = 10 min after apical addition of mixed micelles. Both LD fluorescence and light transmission (light) are shown to visualize cell edges, in x/y planes. The results are representative of three (**a**, **b**) or two (**c, d**) separate experiments

Intestinal frozen sections were stained by Oil red O, a neutral lipid binding dye commonly used to label lipid droplets or lipoproteins (Fig. 1a). No staining was observed in the mucosa of starved mice. In contrast, an accumulation of the dye, corresponding to CLD or chylomicrons, was evident in enterocytes and in the lamina propria of samples harvested 1 or 4 h after lipid gavage. Similar accumulation of CLD takes place in vitro during lipid absorption from apical mixed micelles, for a few minutes to 1 h, as shown using either isolated mouse intestine, stained with red oil O (Fig. 1b) or differentiated Caco-2 cells colored with the fluorescent Bodipy 493/503 or LD540 (Fig. 1c) where the cell outline were indicated by immunodetection of ZO-1 (from tight junctions) and the nucleus by DAPI staining. Fluorescent live imaging of Caco-2 cells, incubated with 1 μg/ml of LD540, showed no intracellular droplets before lipid loading (controls T = 0 min, Fig. 1d). When mixed micelles were added to the apical milieu, CLD accumulated which progressively settled in the cell basement as shown at T = 10 min after lipid addition. However, before settling, the intracellular movement of CLD were too fast to be experimentally detected.

**Fig. 2:**
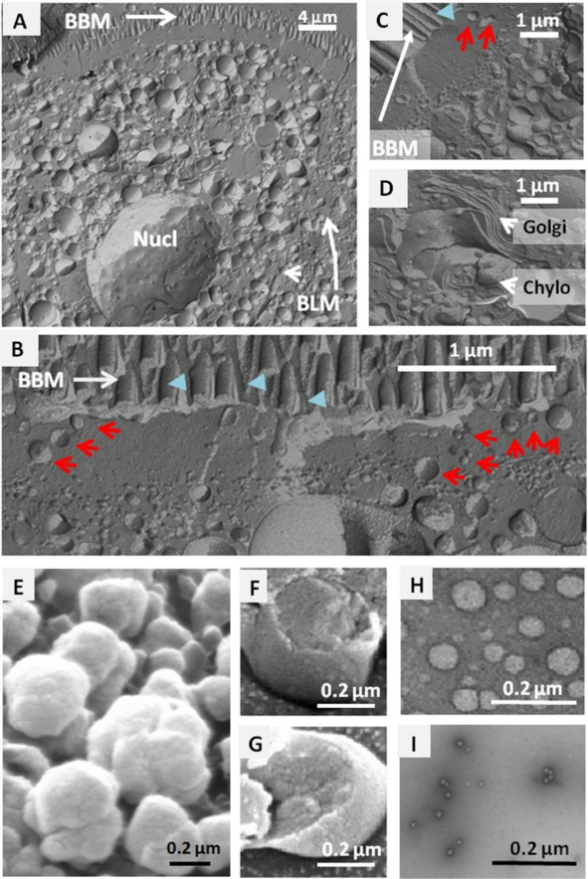
Electron microscopy analysis of CLD. SEM localization of CLD in freeze fractured enterocytes collected at 4 h after lipid gavage (**a**–**d**): CLD in (**a**) the entire cell or (**b**, **c**) the cytosol showing they abundance and size heterogeneity; (**d**) Golgi apparatus (Golgi) and chylomicrons (Chylo) in the secretory apparatus. Several CLD with characteristic lipid monolayer were present in the sub-apical terminal web of enterocyte (red arrows) while BBM had bilayer membranes (blue arrow heads). Observation of purified CLD (**e**–**i**), SEM views of D1 before (**e**) or after freeze fracture (**f**, **g**), and TEM sights of D2 (**h**) and D3 (**i**) fractions. BBM: Brush Border membrane, BLM: Basolateral membrane, Nucl: Nucleus, Golgi: Golgi apparatus, Chylo, Chylomicrons

Freeze fracture experiments were also performed in enterocytes isolated from proximal intestine, 4 h after lipid gavage. The CLD had colonized the whole enterocyte and were heterogeneous in size, from a few to several hundred nanometers in diameter (Fig. 2a–c). One of the major features of freeze fracture is its ability to provide planar views of lipid mono- and bilayers. This helped to separate the CLD (Fig. 2b, c), which are surrounded by an outer lipid monolayer and have a single cross-sectional plane, from the chylomicrons (Fig. 2d), which are enclosed in the membranes of the secretory apparatus with two cutting planes. Some CLD were thereby identified in the sub-apical terminal web region extending 1–2 μm just below the brush border, in an area containing deep apical structures and raft microdomains (Fig. 2b, c).

The CLD from intestinal mucosa were isolated from duodenum and proximal jejunum at 4 h after lipid gavage, corresponding to a maximal accumulation of lipids in both the intestine and plasma [5, 26]. The D1 were subjected to freeze fracture and SEM (Fig. 2e, f, g) showing their outer lipid monolayer and the 3 groups were submitted to TEM after a negative staining of fresh droplets, to assess their sizes and purity (not shown for D1, Fig. 2h, i). The D1 droplets had a similar size to that of chylomicrons and were the most abundant, accounting for 6 % of total intestinal proteins (Table 1), while D2 droplets were ten times smaller. The heterogeneity and scale of sizes measured for isolated D1 and D2 matched those observed in enterocytes after freeze fracture (Fig. 2b–c). The denser D3 droplets were barely detected by TEM, the largest having a diameter of about 13 nm (Fig. 2i), as are plasma HDL. All three populations were authenticated by electron microscopy as coated by a single characteristic lipid monolayer and devoid of any membrane contamination either outside or in their core. During the homogenization of the tissue, lipoproteins present in the secretory apparatus remained mostly trapped in microsomes, and were pelleted with other membranes in the first ultracentrifugation at 100 000xg for 1 h, being therefore separated from the CLD.

**Table 1:** Principal characteristics of intestinal CLD, D1, D2 and D3

CLD	Centrifugation	d (g/ml)	Size (mean diameter ± SD)	Protein content in % of total in I
D1	20,000 g 30 min	1	793 ± 409 nm (n = 40)	6 %
D2	100,000 g 2 h	1	63 ± 42 nm (n = 39)	1 to 3 %
D3	120,000 g 14 h,	1.21	13 ± 4 nm (n = 25)	0.5 to 1 %

Is noted the speed of the last centrifugation performed to overload the droplets, the density of the solution used, the size of the droplet measured by TEM and the percent of protein content in the droplets compared to the total content in the initial homogenate

### Protein analysis of CLD and intracellular localization

**Fig. 3:**
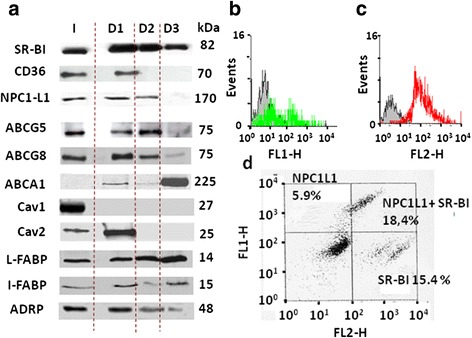
Protein immuno-detection in intestinal CLD by Western-blot and cytometry. (**a**) Western-blot analysis in the total homogenate I or in the droplets D1, D2 or D3 showing discrete expression of SR-BI, CD36, ABCG5, G8 and A1, caveolin (cav) 1 and 2, and the I- and L-FABP. Equivalent protein amounts were loaded for each sample and proteins molecular weights are noted in kDa. (**b**, **c**, **d**) Immuno-detection by flow cytometry of NPC1L1, SR-BI and NPC1L1 plus SR-BI, respectively, in D1 droplets already fixed with PFA. Bars: 10 μM

Intestinal CLD were characterized by Western-blots to identify their associated proteins. A previous study identified the lipid transporter SRBI, which is mainly expressed in the enterocyte BBM, to be transferred to CLD produced after an apical lipid load [11]. Consistent with that, we found SR-BI present in all D1, D2 and D3 populations using three monospecific antibodies (Fig. 3a). When looking to other apical transporters, CD36 was selectively identified in D1, while NPC1L1 was found in D1 and D2, but not in D3. Surprisingly, ABCG5 and G8, which are known to mediate apical efflux but not absorption of sterols, were also detected in D1, D2 and, to a lesser extent, in D3. Finally, ABCA1 remained below the detection limit in the total homogenate (I), was barely detectable in D1 and D2, but was strongly enriched in the D3 droplets. Similar results were obtained with the different antibodies tested which include at least one immuno-specific antibody/transporter. Among these transporters, NPC1L1, SR-BI, ABCG5, G8 and CD36 were all predominantly localized in the BBM of proximal intestine (Additional file 1: Figure S1, [5, 6, 10, 11, 37], while ABCA1 seems broadly distributed in enterocytes [38]. Interestingly, the distribution of lipid transporters clearly distinguished the three intestinal CLDs as having unique protein profiles.

Among mammalian markers of CLD, previous studies identified perilipin 2 (ADRP) and 3 (Tip47) in enterocyte CLD [15, 36] and we detected ADRP in D1, D2 and D3 [Fig. 3a, 26]. Caveolin 1 and 2 were also analyzed which are frequently identified in CLD [22, 23] and are abundant in the enterocyte BBM [39, 40]. Caveolin 1 was found absent from our intestinal CLD. By contrast, caveolin 2 was selectively localized in D1 (Fig. 3a) suggesting its involvement in lipid absorption in mammals, as described earlier in *Caenorhabditis elegans* [40]. Moreover, the I- and L-FABP, which are involved in the cytosolic traffic of lipids from the BBM to the ER [14, 15], were identified in D1, D2 and D3, consistent with a similar detection in enterocyte CLD by proteomics [27]. Our results identified in the CLD several proteins such as membranous transporters, caveolin 2, the cytosolic I-and L-FABP which were all shown involved in intestinal lipid absorption as well as the CLD marker ADRP.

The presence of SR-BI and NPC1L1 at the surface of D1, the only droplets large enough to be detected by this technique, was confirmed by flow cytometry (Fig. 3b–d). Only a subpopulation of 36 % of D1 was specifically immuno-positive for NPC1L1, 28 % for SR-BI with a strong signal (Fig. 3b, c). Interestingly, a similar experiment showed that NPC1L1and SR-BI were co-expressed in 18 % of the D1 population, while only NPC1L1 or SR-BI was detected in respectively 6 % and 15 % of them (Fig. 3d). Note that in a given cell, the differential localization of proteins in some subpopulations of CLD is a common observation.

**Fig. 4:**
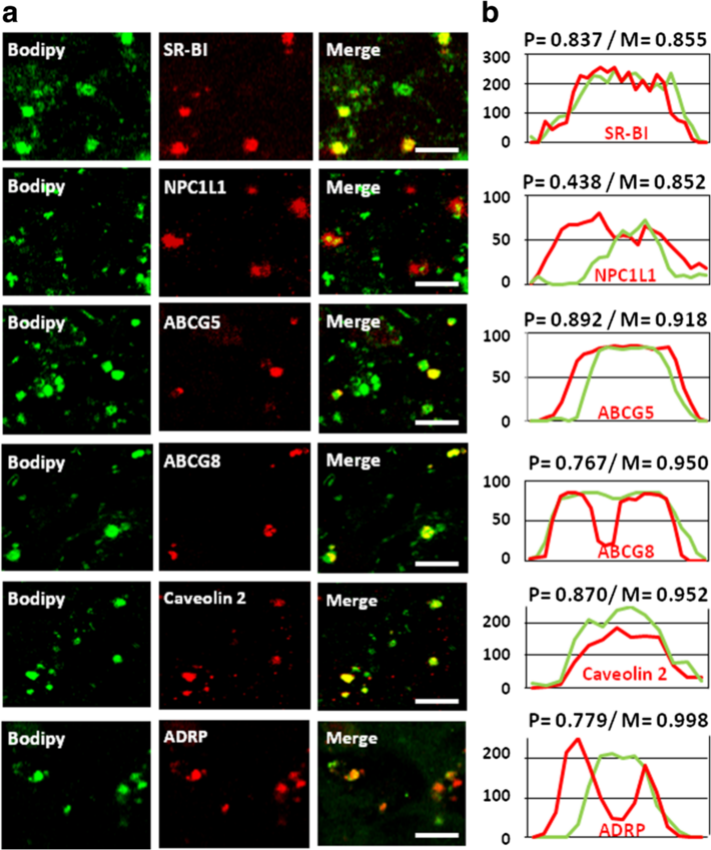
Protein immuno-fluorescent detection in CLD from Caco-2 cells. (**a**) Confocal immuno-detection of proteins in the CLD of differentiated Caco-2 cells fixed with PFA after 10 min of lipid absorption from apical micelles. Green: Bodipy 493/503 staining; Red: specific immuno-detection of either SR-BI, NPC1L1, ABC-G5, −G8, caveolin 2 and ADRP. Bars: 10 μM. These results are representative of three separate experiments. (**b**) The signals from the protein immuno-detection and Bodipy have Pearson’s coefficient correlations (P) and Mander’s overlaps (M) indicating high degrees of correlation. Intensity profiles were generated along linear zones of interest across a CLD indicating that the signal of each protein (red) overlaps or flanks the Bodipy signal (green)

To verify if these transporters are truly located on CLD, we performed confocal immunofluorescent microscopy on Caco-2 cells, fixed 10 min after an apical addition of lipids. The technique allows the detection of large intestinal CLD with diameters of several hundred nanometers, matching D1, in which we identified SR-BI, NPC1L1, ABCG5, ABCG8, caveolin 2 and ADRP in selective subpopulations (Fig. 4a). They showed overlapping or flanking fluorescent intensity profiles compared with Bodipy and high Pearson’s correlation and Mander’s overlap coefficients, indicating high degrees of colocalization (Fig. 4b).

### CLD form underneath the brush border of Caco-2 cells within seconds of lipid load

**Fig. 5:**
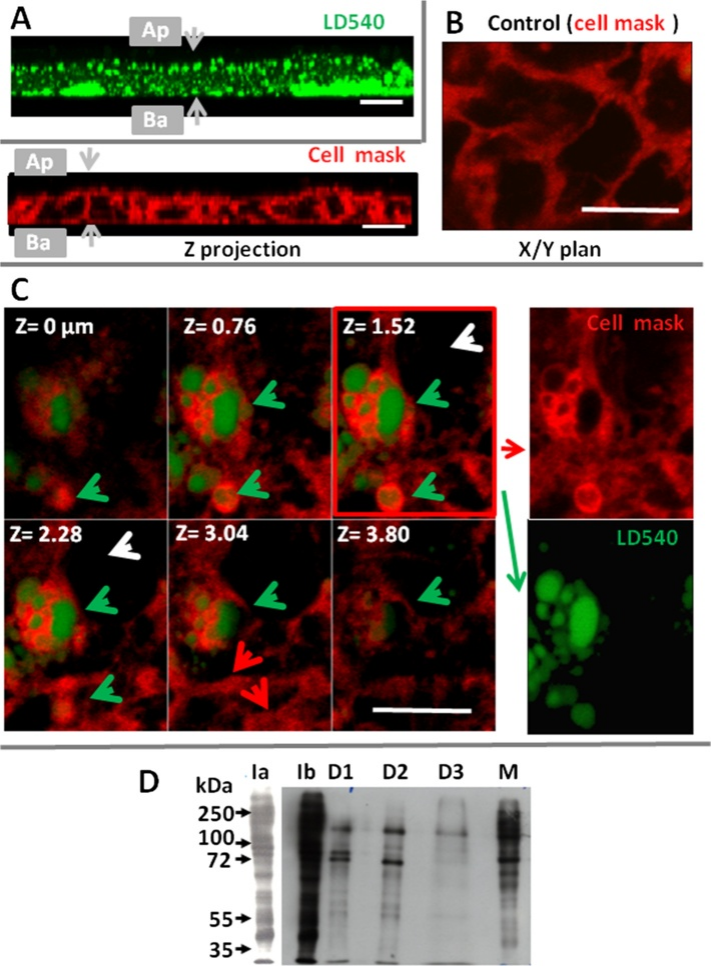
Relationship between CLD, the BBM and lipid metabolism enzymes studied in Caco-2 cells (**a**, **b**, **c**) or in mouse intestine (**d**). Caco-2 cells were stained with cell mask (**b**, **c**), then incubated for 10 s (**a**), 5 s (**c**) with apical mixed micelles, then fixed with EDAC and incubated with the LD540 dye to stain CLD (shown in green). **a**) Z-stack showing nascent CLD situated just underneath the BBM (Z = 0 μm) at upper left position to deeper in the cytosol at lower right. **b**) In control cells, only plasma membranes are labelled by the Cell mask TM (red staining) and intracellular lipid droplets are absent. **c**) After 5 s of an apical lipid load, Cellmask is present in plasma membranes (red arrows) but also coating nascent intracellular CLD (green arrows) but is not internalized in the cytosol of cells devoid of CLD (white arrows), Bar: 10 μM. **d**) Detection of apical biotinylated proteins in the I homogenate (Ia, Ib), in D1, D2, D3 and in M (membranes). Ia and Ib are two time chemiluminescent exposures of the same sample. Equivalent amounts of proteins were loaded and molecular weights are noted in kDalton (kDa). The experiments shown are representative of two separate experiments

We then analyzed the relationship between the apical membrane and CLD immediately after lipid load. Caco-2 cells were stained with CellMask™ Deep Red (Fig. 5a–c), and exposed to mixed micelles for 5 or 10 s of contact, and fixed by 0.5 % of EDAC, a protein cross-linking agent that caused a virtually instantaneous and irreversible cell fixation. As shown in Fig. 5a, numerous CLD, stained by LD540 (revealed in green), were selectively identified after only 10 s of contact with micelles, the fixative EDAC showed a widespread distribution of CLD in enterocyte cytosol but also their enrichment in the sub-apical location, which were not observed after PFA fixation. These results show that intestinal CLD were very quickly produced during fat digestion, just underneath the BBM. Control cells received no lipids and presented a labelling of plasma membranes by the Cell mask dye (artificially shown in red) but the absence of any intracellular CLD (Fig. 5b). An even shorter time of 5 s of contact with micelles was sufficient to detect in several enterocytes an internalization of the cell mask dye and LD540 (shown in green) which were absent in other adjacent cells (Fig. 5b). In separate pictures (Fig. 5c  right pannels), both dyes clearly appeared in discrete cytosolic localizations, with a peripheral staining with Cell mask overlying central structures filled with neutral lipids, and stained with LD540, the whole forming a unit very similar to CLD. This strongly suggests a very fast transfer of lipid constituents from the apical membrane, surrounding nascent lipid droplets.

### Transfer of apical proteins to CLD in mouse intestine

We also analyzed whether apical proteins could be transferred to CLD, during lipid absorption. For that purpose, the apical proteins of isolated mouse intestines were labeled with biotin. Tissues were then incubated with luminal lipid micelles, followed by tissue homogenization and CLD isolation. As shown in Fig. 5d, several biotinylated proteins were detected in D1, D2 and D3 showing clearly distinct profiles as compared to the homogenate I or to plasma membranes and microsomes recovered in M. The patterns of biotinylated proteins were similar in D1 and D2, except for a 90 kDa protein selectively enriched in D1, but distinct from the D3 pattern.

### Proteins involved in lipid metabolism are detected in the apical membrane of mice enterocytes

It is well established that the genesis of CLD takes place at the ER of eukaryotic cells where the major enzymes responsible for the synthesis of triglycerides and cholesteryl ester (MGAT2, DGAT1, and ACAT2) reside [1, 3, 41–43]. In enterocytes, several proteins involved in lipid metabolism or transfer and chylomicron synthesis in the ER, have also been localized in lipid droplets, such as MGAT2 and MTTP [26–28], but also, more surprisingly, in the BBM of enterocytes for MTTP [44].

**Fig. 6:**
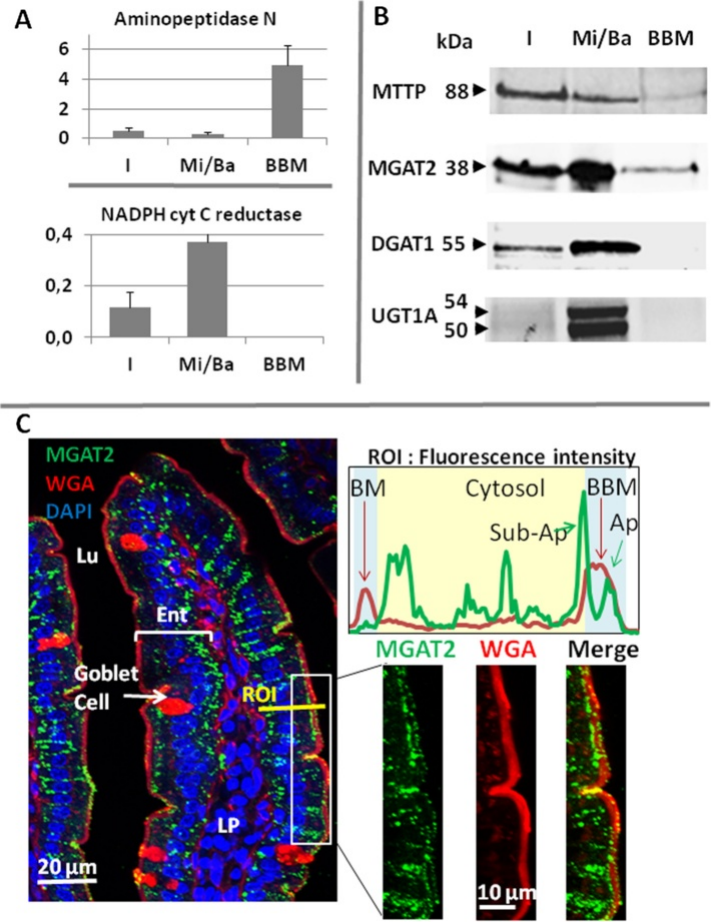
Distribution of metabolic enzymes in mouse proximal intestine. **a**) Enzymatic activities (Units/mg of protein) of aminopeptidase N and the NADPH cytochrome C reductase, in the I homogenate, the Mi/Ba membranes and the BBM of mouse enterocytes : means ± SD of three separate experiments. **b**) Detection by Western-blots of MTTP, MGAT2, DGAT1 and UGT1A in the same three samples. For the electrophoresis, equivalent amounts of proteins were loaded and molecular weights are noted in kDalton (kDa). **c**) Immuno-fluorescent detection of MGAT2 (green) in proximal jejunum associated to membrane staining with WGA- Alexa Fluo 594 (red) which permitted a clear localization of the BBM and to DAPI (blue) for nucleus identification. The merge images showed a spread and punctuated distribution of MGAT2, from the basolateral (BM) to the sub-apical membranes, but also inside the BBM. To underlight this dual localization the intensity of fluorescence of MGAT2 and WGA were quantified in a ROI crossing an enterocyte. Ent: enterocytes, Lu: lumen, LP: lamina propria. The experiments shown are representative of 3 (**a**, **b**) or 2 (**c**) separate experiments

We therefore assessed the protein distribution of MGAT2 and DGAT1, being the two major enzymes metabolizing diet fatty acids into DG and TG in enterocytes. We isolated the enterocytes of fasted mice and separated BBM and Mi/Ba membranes by Mg^2+^ precipitation and sequential centrifugations. As expected, aminopeptidase N, an apical marker, was preferentially found in the BBM, whereas the ER specific NADPH cytochrome C reductase was overwhelmingly recovered in microsomal and basolateral membranes (Fig. 6a), showing an efficient separation of the two types of membranes. DGAT1 and two isoforms of the ER resident UDP glucuronosyltransferase 1 family (UGT1A) were only detected in the Mi/Ba fraction by western-blot. By constrat MTTP and MGAT2 were clearly identified in the Mi/Ba membranes, but also in the BBM (Fig. 6b). The immuno specificity of the anti-MGAT2 used were verified using intestine of mice deficient in the enzyme (Additional file 2: Figure S2)

We additionally performed MGAT enzymatic assays and showed that both types of membranes were producing DG and TG from mono-oleoylglycerol and oleoyl-CoA, confirming the presence of MGAT activity in both samples (Table 2).

**Table 2:** MGAT enzymatic assays using 100 μg of proteins from either microsomal/basolateral membranes (Mi/Ba) or BBM

	Mic/ Ba	BBM
Lipids	nmol/mg	nmol/mg
DG 16–16	11,23	4,22
DG 16–18	35,80	11,83
DG 18–18	0,00	8,69
DG18–20	0,00	0,00
Total DG	47,03	24,73
Chol-C16	14,33	0,00
Chol-C18	29,21	0,00
Total CE	43,54	0,00
C53-TG (16/16/18)	60,54	1,51
C55-TG (16/18/18)	118,91	4,25
C57-TG (18/18/18)	104,93	16,67
C59- TG (18/18/20)	13,58	2,05
Total TG	311,22	24,48
Total DG + TG + CE	401,79	49,21

The assay measured the production of diacylglycerols (DG), cholesterol ester (CE) and triglycerides (TG) in nmoles/mg of proteins, by the membranes incubated for 90 min at 22 °C in the presence of 50 μM of mono-oleoylglycerol and 50 μM of oleoyl-CoA

Immuno-histological microscopy allowed us to further localize the enzyme MGAT2 in the proximal intestine (Fig. 6c) A co-staining of membranes with the lectin WGA-AF594 clearly emphasized the BBM enrichment in glycosylated proteins at the surface of intestinal villi. MGAT2 showed a punctate and wide-spread distribution throughout the enterocytes cytosol consistent with ER localization, but was also detected inside the apical membrane as small dots. Measuring the fluorescence intensity in a region of interest (ROI) clearly showed the specific labeling of basolateral membrane (BM) and BBM by WGA and confirmed the presence of MGAT2 in the cytosol from BM to BBM, but also identified two MGAT2 peaks associated with the sub-apical and the apical region inside BBM.

## Discussion

We recently identified and characterized CLD populations generated in mouse intestine following lipid gavage and isolated by differential density gradient [26]. Our method allows isolation of three subpopulations of lipid droplets of different sizes, from a few microns for D1, to about 60 or 10 nm, respectively, for D2 and D3, which must globally gather most intestinal CLD. In this study, we analyzed the structure, protein composition and the genesis of these CLD. Our results show a close link between apical BBM and a very fast production of CLD enriched in apical proteins, suggesting a role of CLD in the intracellular routing of dietary lipids.

Indeed, at 4 h after lipid gavage in mouse, we identified that the main lipid transporters of the apical membrane, including SR-B1, CD36, NPC1L1, ABCG5/G8 were differentially transferred in D1, D2 and D3 together with the absorbed lipids (Fig. 3) as well as several proteins apically biotinylated (Fig. 6c). The Caco-2 cellular model was also used to reproduce several aspects of lipid absorption including apical uptake, CLD production and chylomicron synthesis and secretion. It permitted us to showed with an additional technique that the lipid transporters SR-B1, NPC1L1, ABCG5/G8 are relocated on the Caco-2 CLD formed after apical lipid load, as is the plasma membrane dye Cellmask (Fig. 6a, b). This shows a transfer of proteins and lipids from the BBM to the droplets. Using isolated proximal intestine, we similarly detected such an internalization of several biotinylated brush border proteins to CLD.

The presence of integral membrane proteins in CLD was unexpected but is not a contamination by membranes in contact or inserted in the core of CLD. Indeed, we found very low membrane contamination in D1, D2 and D3 as assessed by the absence of caveolin 1 or the very low level of apical BBM markers (this study and 26), but also from the protein profiles and notably the ABCA1 enrichment in D3 and the distribution of biotinylated proteins. Relative levels of lipid transporters, caveolin 2 or FABP in CLD, in proportion to the content of the original homogenate, are 10 to 50 times greater than that of other membrane markers (aminopeptidase, alkaline phosphatase or NADPH cytochrome C reductase), suggesting a true expression rather than contamination. This suggests that the purification by flotation may exclude the rise of droplets enriched in membranes.

The list of membrane proteins identified in CLD obtained from many cell types or organisms continues to expand and includes CD36, SR-BI, MGAT2, MTTP, lysophospholipid acyltransferase 2, short chain dehydrogenase/reductase 3, the 7-dehydrocholesterol reductase, squalene monooxygenase, the sodium/potassium-transporting, ATPase subunits alpha-1 and beta-1, prostaglandin E2 or leucotriene-C4 synthases and NS4B [11, 26, 29, 30, 32, 44–48]. Proteomic studies have rarely described lipid transporters, contrary to the methodologies we used in this report, to analyze our CLD populations.

The lipid transporters involved in the intestinal uptake of lipids are not strictly mono-specific but can recognize and bind several liposoluble ligands (fatty acids, sterols and liposoluble vitamins). They were all identified in CLD with differential association: CD36 which is mainly involved in the absorption of fatty acids but also facilitates the uptake of cholesterol in the proximal intestine and the overall production of chylomicrons [4, 8, 49], was selectively identified in the TG-rich D1. The main transporter of cholesterol, NPC1-L1 [2] was detected in both D1 and D2. Its deletion in mice lead to 70 % reduction of cholesterol absorption but not that of fatty acids [1, 6]. Moreover the selective inhibition of NPC1L1 by ezetimibe decreases the internalization of the receptor and cholesterol, the production of cholesteryl ester in the intestine and its release to the lymph [50, 51]. Finally, although the contribution of SR-BI to lipid uptake is more controversial, suggesting that it is not essential but rather facilitating the process [1, 2, 5, 9], the transporter was found in the three CLD populations showing its involvement in the process (Figs. 3, 4).

The presence of ABCG5/G8 and ABCA1 on CLD was not expected, given that they usually export their substrate out of the cells. For instance, ABCG5/G8 contributes to the apical retro-efflux of sterols (phytosterols, cholesterol) neo-absorbed by enterocytes [52]. However, in eukaryotes, a direct requirement of ABC-transporters has recently been established not only in the influx of sterols in yeast and toxoplasma but also in the uptake of abscisic acid and auxin in plants [17, 53–56]. Moreover, consistent with the predominant expression of ABCG5/G8 that we found in D1 and D2 which are rich in TG, the ABCG5 or G8 deletion in mouse causes a significant decrease in the intestinal uptake of both cholesterol and TG but also their secretion in the lymph [57, 58]. In addition, the D3 droplets are strongly enriched in ABCA1, cholesterol and cholesterol ester but low in FA, consistent with the ABCA1 deficiency decreasing the uptake of cholesterol (but not FA) by 28 %, resulting in an inhibition of cholesterol secretion to HDL but not to chylomicrons [59].

These different distributions may reflect a specialized function for the different CLD and for lipid transporters in the intracellular trafficking and exchange of lipids in enterocytes from the BBM to CLD.

Thus, ABCG5/G8, NPC1L1, SR-BI and CD36 are mainly detected in the D1 and D2 droplets and contribute to lymphatic delivery of lipids, linking these CLD to a cytosolic traffic dedicated to chylomicrons. By contrast ABCA1 and D3 appear as good candidates for the intracellular trafficking dedicated to HDL.

MGAT2 and DGAT1 are the major ER enzymes involved in the synthesis of TG in enterocytes, a step absolutely required to the genesis and the growth of CLD and of chylomicrons [30, 31, 34, 40, 41]. As expected we identified them by membrane fractionation and western-blot as predominantly expressed in the microsomal fraction containing ER membranes, together with MTTP and UGT1A. However, we additionally detected MGAT2 and MTTP in the fraction containing purified BBM (Fig. 6), where DGAT1 and UGT1A were absent. This dual localization in the ER and the BBM has already been established for MTTP and its protein disulfide isomerase subunit [44]. We additionally confirmed both apical and intracellular MGAT2 in isolated membrane fractions by measuring MGAT enzyme activity and by immuno-histology. Thus, if in most cell types proteins insuring lipid metabolism are mostly expressed in the ER, MGAT2 and MTTP are additionally located in the BBM and the CLD of enterocytes [26]. Interestingly, the caveolae of adipocyte plasma membrane were recently shown to produce TG, suggesting these microdomains to be a gateway for fatty acid entry, triglyceride synthesis and the genesis of CLD, where caveolin 1 seems to be transferred [22, 60, 61]. Our results suggest a very similar synthesis of DG or TG in the brush border of enterocytes, via MGAT2 and the formation of CLD possibly from the apical caveolae enriched in fatty acids either directly or by cooperation between the BBM and adjacent ER extensions [62]. Indeed, the ER is known to be broadly distributed in the enterocyte cytosol, which is consistent with the intracellular immuno-labeling of MGAT2 we observed spreading from basal to sub-apical location. In agreement with these findings, the expression levels of MGAT2 have been shown to determine the uptake, in addition to the esterification, of monoacylglycerol generated from the digestion of dietary lipid [63, 64]. In addition, it has been shown that another TG producing enzyme, DGAT2, having a similar structure to MGAT2, is able to synthesize CLD when located in another place than the ER [65]. We thus hypothesize that part of the intestinal CLD could possibly be produced directly from the BBM which is consistent with their very fast production; the apparent transfer of apical transporters in them and the apical expression of MGAT2. This is further supported by the presence of CLD in the sub-apical terminal web of enterocytes, enriched in detergent resistant membrane microdomains where lipids first accumulate during absorption. We therefore proposed that intestinal CLD as possibly involved in the trafficking of lipids between the BBM and the ER which is also consistent with the presence of I and L-FABP and NPC1L1 since they are involved in this process [1, 2, 13–15]. This hypothesis fits well with a recent identification of co-localizations of ABCG transporters and a sterol-O-acyltransferase in membrane microdomains of plasma membranes, facilitating sterol uptake and esterification [18]. The local esterification of sterol, synthesis of TG and genesis of CLD would then prevent the cellular toxicity of free sterol or fatty acids, abundantly absorbed in enterocytes during digestion

In the uptake process, it remains unknown to what extend the internalization of lipid transporters, we and previous studied have detected, is attributable to either endocytosis or CLD production as we suggest. Similarly, it remains to be determined the places and moments of ER intervention, either directly or through membranes contact sites [62] with the apical membrane. Nonetheless, our results suggest that the apical membrane and the CLD may partially metabolize FA in addition to the predominant action of the ER in the process.

## Conclusion

We show that intestinal CLD are produced very quickly at close proximity to the BBM following lipid addition, and could represent the structure insuring cytosolic traffic of lipids to the ER. Several apical proteins seem to be transferred to the CLD including lipid transporters. The enterocytes offer a model of choice to study the cellular absorption of lipids, being polarized and highly absorptive. The present study also provides evidence suggesting that lipid transporters contribute to intracellular lipid conveyance through CLD. This opens new perspectives to decipher the molecular mechanisms of lipid uptake and transfer through enterocytes.

## Abbreviations

ABCG5/G8: ATB-binding cassette transporter G5 and G8; ADRP: adipocyte differentiation-related protein; BBM: brush border membrane; CLD: cytosolic lipid droplets; DG, diacylglycerol; DGAT, diacylglycerolacyl transferase; EC, esterified cholesterol; ER, endoplasmic reticulum; FABP: fatty acid binding protein; FC: free cholesterol; IAP: intestinal alkaline phosphatase; iHDL: intestinal HDL; LD: lipid droplets; MGAT 2: monoacylglycerol acyl transferase 2; MTTP, microsomal triglyceride transfer protein; NPC1L1: Niemann-Pick C1-Like 1 protein; SEM: scanning electron microscopy; SR-BI: scavenger receptor class B member 1; TC: sodium taurocholate; TEM: transmission electron microscopy; TG: triacylglycerol; ZO-1: Zonula occludens-1

## Additional files

Additional file 1: Figure S1. Immuno-histochemical localization of duodenal SR-BI, NPC1L1 and ABCG5 and G8 in the BBM of starvedmice duodenum. Bar:15 um. (TIF 430 kb) Additional file 2: Figure S2. Specificity of MGAT antibody demonstrated by western blotting and immuno-fluorescence microscopy. (a) Immunoblot of jejunumhomogenates from wildtype (WT) and MGAT2-deficient (*Mogat2* ^-/-^) mice. (b) Immunofluorescence of jejunum sections from WT and *Mogat2* ^-/-^ mice. (TIF 329 kb)
