# Supplementary figures and images for: Exposure to dietary lipid leads to rapid production of cytosolic lipid droplets near the brush border membrane
Source: Nutr Metab (Lond). 2016 Jul 28;13:48. doi: 10.1186/s12986-016-0107-9 (PMC4965885; doi:10.1186/s12986-016-0107-9)

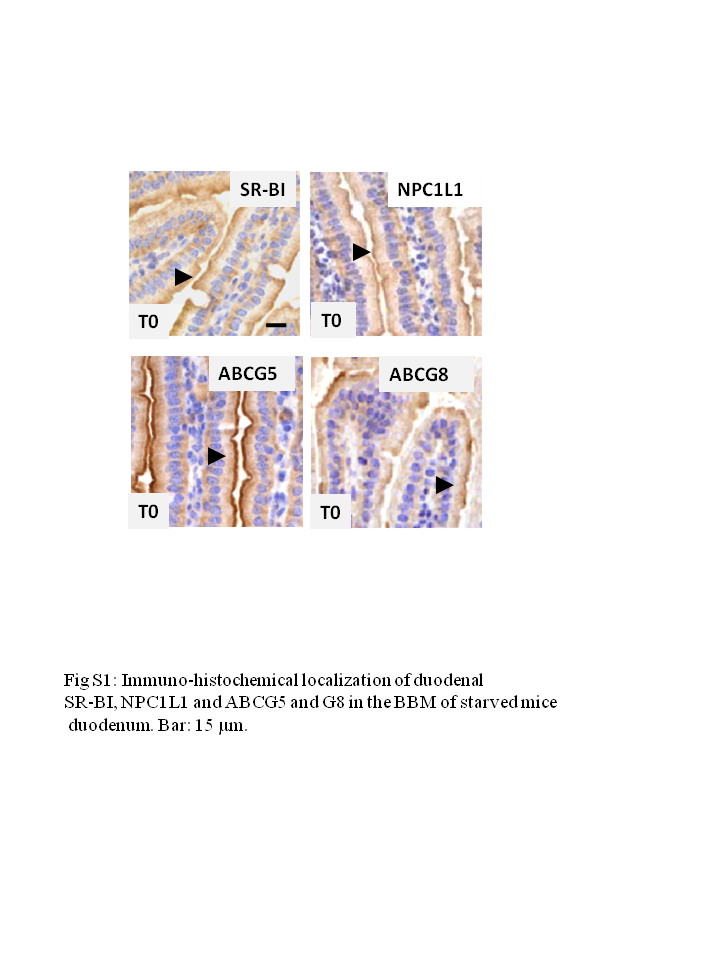

Supplement: Additional file 1: Figure S1. — Immuno-histochemical localization of duodenal SR-BI, NPC1L1 and ABCG5 and G8 in the BBM of starvedmice duodenum. Bar:15 um. (TIF 430 kb) [file 12986_2016_107_MOESM1_ESM.tif]

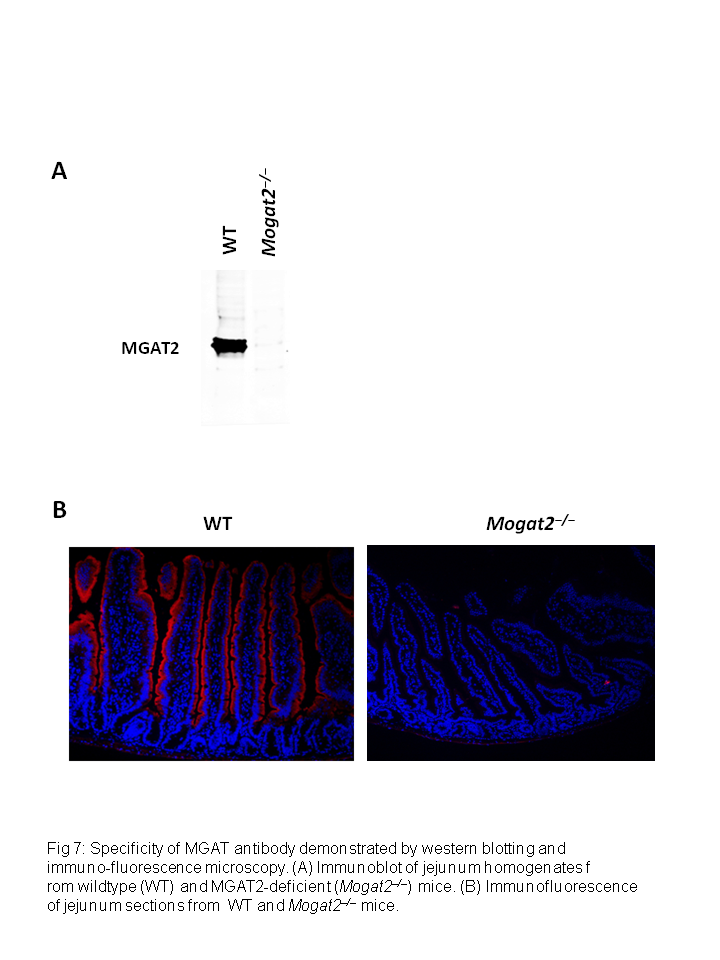

Supplement: Additional file 2: Figure S2. — Specificity of MGAT antibody demonstrated by western blotting and immuno-fluorescence microscopy. (a) Immunoblot of jejunumhomogenates from wildtype (WT) and MGAT2-deficient (Mogat2 -/-) mice. (b) Immunofluorescence of jejunum sections from WT and Mogat2 -/- mice. (TIF 329 kb) [file 12986_2016_107_MOESM2_ESM.tif]
